# Advancing Electrochemotherapy in Melanoma and Non-Melanoma Skin Cancer: From Clinical Practice to Emerging Frontiers

**DOI:** 10.3390/ijms27198440

**Published:** 2026-09-22

**Authors:** Ramona Marrapodi, Arianna Presaghi, Emilia Migliano, Barbara Bellei

**Affiliations:** 1 Laboratory of Cutaneous Physiopathology and Integrated Centre of Metabolomics Research, San Gallicano Dermatological Institute, IRCCS, Via Elio Chianesi 53, 00144 Rome, Italy; ramona.marrapodi@ifo.it; 2 Department of Plastic and Reconstructive Surgery, San Gallicano Dermatological Institute, IRCCS, Via Elio Chianesi 53, 00144 Rome, Italy; arianna.presaghi@ifo.it (A.P.); emilia.migliano@ifo.it (E.M.)

**Keywords:** electrochemotherapy, skin cancers, melanoma, NMSCs, immune modulation

## Abstract

Electrochemotherapy (ECT) is a locoregional treatment that combines poorly permeable drugs, commonly bleomycin (BLM) or cisplatin (CDDP), with electric pulses that induce electroporation. By transiently increasing cell membrane permeability, ECT promotes intracellular drug accumulation, enhancing cytotoxic effects while minimizing undesirable systemic toxicity. Beyond its direct cytotoxic effects, ECT exerts additional antitumour mechanisms that contribute to tumour control. These include the induction of immunogenic cell death (ICD), leading to activation of innate and adaptive immune responses; the “abscopal effect”, achieved with the combination of ECT and immune checkpoint inhibitors; and the “vascular lock” phenomenon, promoting a transient local blood flow reduction and enhancing intratumoural drug retention; and a “vascular disrupting effect” that contributes to tumour vascular damage and secondary tumour cell death. ECT has demonstrated high objective response rates (ORR) across a wide range of cutaneous malignancies, including melanoma and non-melanoma skin cancers (NMSCs) and cutaneous dissemination of other tumours. Although predictive biomarkers capable of identifying patients who benefit from treatment are still lacking, substantial preclinical and clinical evidence supports the efficacy, safety and tolerability of ECT. Several ongoing clinical trials are currently evaluating ECT, particularly in advanced or non-resecable melanoma, aiming to investigate further biological effects and optimize therapeutic strategies. Furthermore, innovative approaches integrating ECT with gene electrotransfer and nanomedicine represent a promising avenue to enhance its therapeutic efficacy and expand its role in precision oncology.

## 1. Introduction

Electrochemotherapy (ECT) has emerged as a highly effective, minimally invasive therapeutic modality for the management of melanoma [1] and non-melanoma skin cancers (NMSCs) [2,3], the two major categories of skin cancer, particularly serving as an alternative treatment option for patients who are not eligible for standard surgical excision alone or are no longer responders to conventional therapies.

NMSCs are the most common group of malignancies worldwide [4]. They are primarily classified according to their cellular origin: squamous cell carcinoma (SCC) arises from the uncontrolled proliferation of keratinocytes within the suprabasal epidermal layers, whereas basal cell carcinoma (BCC) originates from the stratum basale (the deepest epidermal layer) [5,6]. Melanoma arises from the malignant transformation of melanocytes, highly specialized pigment producing cells, primarily located in the basal epidermal layer and fully surrounded by keratinocytes [7]. Although characterized by distinctive biological features and divergent clinical behaviours, both melanoma and NMSCs share ultraviolet (UV) radiation from solar exposure as their primary environmental risk factor [8,9]. Due to their superficial localization, these malignancies offer straightforward surgical accessibility; consequently, complete surgical excision remains the preferred cornerstone therapeutic approach for both diseases, demonstrating high efficacy and frequently proving curative for early-stage disease. Depending on the tumour stage and metastatic dissemination, systemic interventions, such as immunotherapy, targeted therapies, chemotherapy and radiotherapy, are indicated for the management of advanced, metastatic and inoperable cases [5,10]. Despite recent breakthroughs in the therapeutic landscape, skin cancers, particularly melanoma, continue to pose significant clinical challenges that necessitate further improvements in treatment strategies. This is primarily attributable to intrinsic or acquired drugs resistance and immune system evasion. For primary tumours and cutaneous or subcutaneous metastases that are surgically unresectable or refractory to conventional systemic therapies, ECT has emerged as an effective locoregional alternative [11,12].

ECT is a dual-component modality that utilizes electroporation to enhance the intracellular delivery of non-permeant cytotoxic drugs into tumour cells. However, there is growing evidence regarding a concomitant favourable impact on the tumour microenvironment. The application of short, high-voltage electrical pulses transiently permeabilizes the cell membrane, thereby allowing a significantly higher concentration of the therapeutic agent to enter the cytosol. This localized approach exerts potent antitumor effects with minimal systemic toxicity, making it exceptionally suited for treating cutaneous and subcutaneous lesions [13]. In line with European initiatives such as the Joint Action JANE 2, there is a growing momentum to harmonize ECT with other interventional tumor ablative techniques, fostering its broader integration into multidisciplinary oncology protocols, as highlighted by Gehl et al. [14].

The aim of this review is to provide an updated overview of the current state of the art of ECT in cutaneous melanoma and NMSCs, integrating evidence from both preclinical models and clinical studies. Furthermore, highly innovative advancements will be discussed to delineate novel therapeutic avenues.

## 2. Brief Description of Electrochemotherapy Procedure

ECT is a locoregional treatment for solid tumour [15]. Despite ongoing advances in the use of ECT through endoscopy, laparoscopy, and open surgery, ECT is currently widely used treatment to target superficial, easily accessible tumours, making cutaneous and subcutaneous lesions ideal candidates for this approach [16]. By utilizing the principle of reversible electroporation, this therapy combines the administration of low-dose, non-permeant or poorly permeant cytotoxic agents with the localized application of controlled electric pulses to increase intracellular delivery. When cells are exposed to a high-intensity electric field, the interaction between the field and the charged phospholipid bilayer induces the transient formation of nanoscale pores in the plasma membrane [17,18,19]. This temporary increase in membrane permeability facilitates the intracellular accumulation of chemotherapeutic agents, enabling them to reach potent cytotoxic concentrations that trigger programmed cell death. By modulating drug delivery in a site-specific manner, ECT maximizes local antitumor efficacy while minimizing systemic toxicity and preserving the structural integrity of adjacent healthy tissues [20]. However, the utility of ECT is restricted to drugs directed against intracellular targets and, due to the reliance on passive diffusion following pulse application, it is selectively effective for hydrophilic molecules [13]. Consequently, the chemotherapeutic agents most widely employed in ECT are bleomycin (BLM) [21] and cisplatin (CDDP) [22]. Due to their hydrophilic nature, these agents exhibit poor baseline membrane permeability and do not readily cross the lipid bilayer under physiological conditions [23,24]. BLM, a cytotoxic agent used in various anticancer regimens, induces DNA single- and double-strand breaks [25]. Consistently, combining electroporation with either BLM or CDDP has been shown to markedly enhance cytotoxicity in vitro compared to chemotherapy alone [26,27]. These in vitro findings were subsequently validated in vivo across multiple solid tumour animal models, demonstrating a significant improvement in antitumor efficacy following ECT [28,29,30]. More recently, calcium electroporation (CaEP) has been introduced as a novel anticancer modality [13]. Administrated intratumorally using a standardized concentration of 220 mM CaCl_2_, with the injected volume calculated according to tumour volume [31], CaEP enables a massive influx of extracellular calcium into the cytosol. This non-physiological intracellular overload triggers a catastrophic metabolic collapse, as cells exhaust their adenosine triphosphate (ATP) reserves through the hyperactivation of calcium-exporting pumps and severe mitochondrial dysfunction. Acute energy depletion, combined with the activation of enzymatic degradation pathways, ultimately drives selective tumour cell death [32]. Further mechanistic insights by Hoejholt et al. demonstrated that differences in membrane lipid composition between malignant and non-malignant cells influence their physical susceptibility to electroporation, supporting the targeted efficacy of CaEP while sparing surrounding healthy tissues [33].

Clinical evidence indicates that CaEP achieves local response rates comparable to traditional ECT using BLM or CDDP, particularly in cutaneous and subcutaneous lesions [34]. Beyond its therapeutic efficacy, CaEP offers distinct practical and logistical advantages: it is significantly more cost-effective, avoids the strict safety protocols and occupational hazards associated with handling cytotoxic drugs, and utilizes a highly stable compound with straightforward storage requirements [35].

Recent studies have investigated alternative agents to extend the application of ECT beyond conventional chemotherapeutic drugs. Specifically, Michel et al. reported that nisin, a cationic antimicrobial peptide with proven anticancer potential, can potentiate electroporation-mediated permeability and subsequent cytotoxicity [36]. This is achieved by lowering the electric field threshold required for membrane permeabilization and delaying membrane resealing in tumour cells, thereby identifying it as a promising novel adjuvant for ECT protocols. Furthermore, the effect of nisin is not restricted merely to sensitizing cells to electric field exposure; rather, an enhanced, synergistic cytotoxicity of this molecule has been observed when explicitly combined with electroporation [37].

A comprehensive 2025 review on reversible electroporation evaluated both established and emerging cytotoxic compounds for their compatibility with ECT protocols. The study analysed how combining chemotherapy with pulsed electric fields enables drastic dose reductions, thereby minimizing systemic toxicity. While BLM and CDDP remain the primary clinical standards, the authors surveyed alternative candidates, including mitomycin C, doxorubicin, and calcium. Furthermore, the review highlights how therapeutic efficacy depends on the interplay between electric field parameters and the molecular structures of the drugs. Finally, untested chemotherapeutics and natural compounds were categorized based on their intrinsic cytotoxic potential and predicted synergy with electroporation [13].

Electroporation is classified as reversible or irreversible, according to the extent and duration of membrane permeabilization induced by electric pulses [38,39]. In reversible electroporation, membrane pores reseal after the electric pulses, allowing cells to recover structural integrity in a short period. This transient permeabilization facilitates the delivery of functional molecules to target cells, inducing cell death by apoptosis, mitotic catastrophe, or pseudo apoptosis. In contrast, irreversible electroporation occurs when electrical parameters exceed a critical threshold, leading to permanent membrane damage and cell death, resulting in substantial tissue ablation [38,39]. Both reversible and irreversible electroporation can elicit a systemic antitumor immune response, thereby augmenting the efficacy of immunotherapy. ECT promotes the release of damage-associated molecular patterns (DAMPs) into the extracellular space, effectively turning the tumour into an in-situ vaccine [40,41].

In 2006, the first standard operating procedures (SOPs) for ECT were established, providing a formalized framework for its clinical implementation [42]. One of the key aspects of these SOPs is the definition of the optimal drug-to-pulse interval. Specifically, the major recommendations concern the application of reversible electroporation within a defined time window, typically a few minutes following drug injection, to ensure optimal intracellular uptake and maximum therapeutic efficacy. With the progressive dissemination of this therapeutic approach across multiple centres, the need to further harmonize treatment protocols and ensure inter-institutional reproducibility has become increasingly evident. Later, an updated consensus was published in 2018, resulting in the revision of the European Standard Operating Procedures of ECT (ESOPE) for the treatment of cutaneous tumours and skin metastases [43]. These guidelines define the key components of the ECT protocol, including patient and lesion selection, drug choice and route of administration, optimization of the interval between drug administration and pulse delivery, pulse parameters required to achieve reversible electroporation, electrode selection and positioning, and peri-procedural pain management. According to the ESOPE protocol, BLM can be administered either intravenously or intratumorally (Figure 1), depending on the tumour volume, lesion count, and patient-specific risk factors. In contrast, CDDP is administered intratumorally and is generally reserved for the treatment of small lesions.

BLM intravenous administration is followed by a therapeutic window of 8–28 min during which electric pulses should be applied, to ensure that the drug reaches its peak concentration within the tumour vasculature. Conversely, intratumoural injection of BLM or CDDP is recommended immediately prior to electroporation, making it highly suitable for isolated or superficial lesions [42]. Regardless of the route of intralesional drug administration, appropriate electrode selection is crucial to achieve effective electroporation throughout the target volume. Electrode choice should be guided by lesion characteristics, including size, depth, and anatomical location. Linear needle electrodes are generally preferred for smaller lesions, particularly in cosmetically or anatomically sensitive areas, whereas hexagonal needle electrodes are more suitable for larger tumour surfaces. For mucosal tumours, finger electrodes may be advantageous owing to their flexible design, which facilitates accurate positioning, while plate or parallel electrodes are typically used for superficial lesions. As the effective electric field is limited to the area encompassed by the electrodes, multiple sequential applications and electrode repositioning are often necessary to ensure complete tumour coverage, including an appropriate safety margin [43].

Having initially been considered merely a palliative treatment for patients with no other therapeutic options, ECT is now widely recognized as a standard of care, as it has been incorporated into the melanoma guidelines of the National Institute for Health and Care Excellence (NICE) and the European Society for Medical Oncology (ESMO) [44,45].

## 3. Mechanisms of Anticancer Effects of ECT

Traditionally viewed as a tumour-ablative technique, ECT primary functions potentiating anti-cancer drug delivery, leading to cell death. Nevertheless, ECT achieves additional, underappreciated effects that indirectly contributes to tumour reduction. A comprehensive understanding of direct and indirect mechanisms may help optimize the use of ECT in clinical practice.

### 3.1. Direct Effects

At the biophysical level, the application of high-voltage electric pulses alters the transmembrane potential creating transient aqueous pores within the lipid bilayer. This structural permeabilization bypasses standard endocytic pathways, allowing non-permeant hydrophilic cytostatic drugs to directly enter the cytosol via passive diffusion through these newly transiently formed nano-channels [46]. Once inside, due to transient nature of treatment, non-lipophilic drugs accumulate intracellularly inducing immediate and severe damage, leading to mitotic catastrophe and triggering multiple forms of regulated cell death, including intrinsic apoptosis, necroptosis, ferroptosis, pyroptosis, and autophagy-associated cell death, without relying on systemic drug accumulation [47].

BLM is a glycopeptide antibiotic that induces oxidative DNA damage. It forms a complex with metal ions (predominantly iron) and oxygen which generates destructive reactive oxygen species (ROS) such as superoxide and hydroxyl radicals [48]. Free radicals directly attack deoxyribose sugar backbone of DNA, resulting in single- and double-strand breaks. This structural damage effectively halts normal DNA synthesis and cell replication, leading to cell cycle arrest and triggering cell death. CDDP is a platinum-based chemotherapeutic agent that forms covalent bonds with DNA. Upon entering the cell, it undergoes activation via aquation and highly reactive, positively charged molecule formation. The activated CDDP travels into the nucleus and binds covalently to purine bases (specifically guanine and adenine) on the DNA strand. It frequently forms “crosslinks” between adjacent bases on the same strand or between opposite strands. These adducts block replication and transcription, activating cellular checkpoints that lead to programmed cell death [49].

### 3.2. Indirect Effects

-**Immunogenic Cell Death**: ECT is recognised as a potent inducer of immunogenic cell death (ICD) [50], a regulated form of cell death capable of eliciting an antigen-specific immune response (Figure 2) [51].

This immunostimulatory effect has been observed following ECT with both BLM [50] and CDDP [52]. However, the magnitude and nature of the immune response appear to be influenced by several factors, including the tumour cell type and the concentration of the chemotherapeutic agent used during treatment [53]. Recently, Bendix et al. investigated the direct, in vitro effects of ECT, on the biology of murine dendritic cells (DCs), key antigen-presenting cells (APC), central to antitumor immunity. The authors exposed murine bone-derived DCs to different electric field strengths (800, 1000, and 1300 V/cm) in the presence of chemotherapeutic agents to evaluate how ECT modulates cell viability, phenotypic activation, and functional maturation [54].

Despite reduced viability at higher voltages, surviving DCs displayed upregulated expression of MHC class II and the co-stimulatory molecule CD80 and altered expression profile of multiple TLRs. Specifically, TLR6 and TLR9 were significantly increased, whereas TLR4, TLR7, and TLR8 levels decreased following treatment. Taken together, the study shows that electric field strength critically shapes DCs survival and function, with lower voltages better preserving functional competence and higher voltages inducing activation at the expense of viability [54].

In line with these observations, the tumour cell damage promotes the emission of endogenous alarm signals, commonly referred to DAMPs [55], which implies tissue stress to the immune system recruitment. The release of these molecules contributes to the establishment of a pro-inflammatory microenvironment that, within minutes after ECT, becomes characterized by the recruitment of both innate [56,57] and adaptive immune cell populations [58,59]. DAMPs help in reprogramming the local microenvironment from an immunosuppressive status into an immunostimulatory environment. Specifically, these signalling pathways enhance the maturation of DCs which phagocyte tumour-associated neoantigens released during ECT and migrate to draining lymph nodes, where they cross-present these antigens to naive T cells. ICD-associated DAMPs are ATP and High Mobility Group Box 1 (HMGB1) protein and endoplasmic reticulum chaperone calreticulin (CRT) proteins [53]. These molecules attract immune cells, including macrophages and neutrophils, to the tumour site, promoting an inflammatory response.

Evidence supporting the role of the immune microenvironment in determining the clinical efficacy of ECT was already suggested by earlier translational studies. In patients with cutaneous melanoma metastases, the distribution and phenotype of tumour-infiltrating lymphocytes (TILs) are closely associated with treatment outcomes [60]. Specifically, a higher CD8^+^ T-cell infiltrate correlates with an improved local response, whereas FOXP3 expression on melanoma cells is linked to an increased risk of visceral progression. These findings indicate that local immunosuppressive mechanisms might significantly influence the effectiveness of ECT [61]. Consistently, Di Gennaro et al. (2016) reported a post-treatment reduction in CD4^+^FOXP3^+^ regulatory T cells alongside an increased infiltration of CD3^+^CD8^+^ cytotoxic T lymphocytes in melanoma metastases, signalling a definitive shift toward a more immunostimulatory tumour microenvironment [62]. These findings provide the hypothesis that ECT may reduce local immunosuppression while enhancing effector T-cell recruitment, thereby contributing to the antitumor immune response in addition to its direct cytotoxic activity. However, despite robust local immune activation, systemic antitumour effects remain inconsistent, providing the rationale for combining ECT with immunotherapeutic approaches [55,63,64]. Therefore, future efforts should focus not only on combining ECT with immunotherapy but also on tailoring treatment strategies according to tumour histology and the immune landscape of the tumour microenvironment, which are likely to influence treatment responsiveness [65]. A major knowledge gap in the field concerns the kinetic optimization of combination regimens specifically, the sequential order of ECT and immunotherapeutic agents alongside the intervening time intervals. Unravelling whether priming the tumour microenvironment with ECT prior to immune checkpoint inhibition yields superior therapeutic efficacy compared with concurrent or reverse dosing schedules represents an urgent avenue for future clinical trials. Recent findings from the multicentre International Network for Sharing Practices of ECT (InspECT) registry have highlighted the importance of treatment timing in combined ECT and immunotherapy. A cohort of 170 patients with stage IIIC–IV melanoma was retrospectively analysed to assess toxicity and local, systemic, and long-term outcomes following combined ECT and immunotherapy. Patients were stratified according to whether immunotherapy was administered before, concomitantly with, or after ECT. The most favourable responses were observed when ECT was performed during ongoing immunotherapy, suggesting that the timing of ECT relative to immune checkpoint inhibition may influence treatment efficacy and potentially enhance the clinical benefit of immunotherapy [66]. These findings are consistent with previous observations by Campana et al., who reported improved local and systemic outcomes with ECT combined with pembrolizumab compared with pembrolizumab alone in patients with cutaneous melanoma metastases [67]. Recent evidence indicates that the induction of ICD may also depend on treatment-related factors, including electroporation parameters, highlighting the need to optimize ECT protocols not only for cytotoxic efficacy but also for their immunomodulatory potential [68]. This study demonstrated that high-frequency reversible electroporation combined with BLM significantly enhanced tumour cell killing and induced hallmarks of ICD, including the release of ATP, HMGB1, and CRT, ultimately promoting CD8^+^ T-cell infiltration and inhibiting tumour growth in vivo.

-**Abscopal effect**: When combined with immune checkpoint inhibitors (e.g., anti-PD1 and anti-CTLA-4), ECT potentiates antitumor immunity sometimes leading to an “abscopal effect”.

This phenomenon, first described as a mechanism associated with radiotherapy [69], refers to the immune-mediated regression of distant, untreated metastatic lesions following a localized therapeutic intervention and is considered a manifestation of systemic antitumor immunity extending beyond the electroporated tumour site [65,70]. Therefore, immunogenicity generated by ECT does not seem to be robust enough to promote abscopal effects and researchers are looking to combine it with immune co-treatments. Interestingly, a recent case report described a patient with Merkel cell carcinoma who, despite experiencing extensive disease progression following ECT, showed a rapid and remarkable response to subsequent avelumab treatment, ultimately achieving a complete response [71]. Furthermore, the combination of BLM electrotransfer and CaEP has been shown to enhance both bystander and abscopal effects in preclinical models, suggesting that the immunological outcome of electroporation-based therapies can be modulated by treatment design and may be further optimized to promote systemic antitumour immunity [72].

-**Vascular block and vascular disrupting action**: Beyond its direct cytotoxic effects on tumor cells, ECT induces distinct vascular effects that contribute to tumour control [60,73]. Tumour vascular architecture inherently dictates both metabolic activity and systemic drug distribution. The application of electric pulses induces an immediate, reflex-mediated vasoconstriction of tumour-feeding arterioles, which is rapidly followed by the structural disruption of the endothelial cytoskeleton and subsequent endothelial cell swelling [60]. This cascade results in a prolonged shutdown of local blood flow, a phenomenon known as the “vascular lock” effect, which physically entraps the chemotherapeutic agent within the tumour bed for several hours. This transient vascular response is not restricted to tumour vessels and may also occur in normal vasculature [74]. A second, delayed mechanism is represented by the vascular disrupting effect, which is associated with damage to the tumour endothelium. While electroporation alone induces transient alterations in tumour perfusion, the presence of BLM enhances endothelial injury, leading to progressive impairment of tumour vessels and, ultimately, their destruction. The resulting persistent reduction in blood flow deprives surviving tumour cells of oxygen and nutrients, thereby contributing to secondary ischemic cell death [60]. Importantly, these vascular effects display a degree of tumour selectivity. An interesting comparative study by Markelc et al. demonstrated a more pronounced electroporation effect on tumour blood vessels than normal blood vessels further encouraging the clinical practice [75]. The greater susceptibility of tumour-associated blood vessels to electroporation may be related to their structural and functional abnormalities, including disorganized architecture, increased permeability, and immature endothelial organization [75].

### 3.3. Predictive Biomarkers

Despite the high response rates achieved with ECT, no validated biomarkers are currently available to reliably predict which patients will benefit from this treatment. This underscores a significant unmet clinical need for robust predictors of tumour response to ECT. Addressing this gap will require systematic investigation of both intrinsic tumour cell characteristics and the tumour stromal compartment, which are likely to jointly influence treatment sensitivity by modulating drug uptake, electroporation efficiency, and microenvironmental dynamics. A more comprehensive characterization of these putative determinants could ultimately refine patient stratification, enable the optimization of treatment parameters, and support the rational development of novel therapeutic combinations [60].

Recently, a preclinical study has investigated the potential role of BLM hydrolase (BLMH) expression in tumour tissue as a determinant of ECT efficacy, providing mechanistic evidence that intracellular drug metabolism may significantly influence treatment response [76]. Since BLM is the primary cytotoxic agent used in ECT, its effectiveness depends not only on electroporation-mediated intracellular delivery but also on its intracellular stability and persistence. In this context, BLMH, an enzyme responsible for the enzymatic inactivation of BLM, may critically modulate its cytotoxic potential. The study demonstrates that high BLMH expression reduces tumour sensitivity to BLM-based ECT, supporting its role as a predictive biomarker [76]. Conversely, lower BLMH expression correlates with improved treatment response, supporting the concept that tumour-intrinsic metabolic activity contributes to inter-tumour heterogeneity in ECT outcomes. Overall, these findings identify BLMH as a putative predictive biomarker of ECT response; however, its clinical applicability remains to be validated in independent and clinically representative cohorts [76]. Although further validations are needed to support the potential targeting of BLMH, the use of this marker in patient selection could help in clinical practice to identify patients who might derive the greatest benefit from ECT.

In addition to metabolic determinants of drug inactivation, stress-response pathways have also been implicated in modulating tumour susceptibility to ECT. In this context, a preclinical study in MDA-MB-231 triple-negative breast cancer cells reported a significant downregulation of key heat shock proteins (HSPs) following CDDP-based ECT [77]. Given the central role of HSPs in maintaining cellular proteostasis and promoting survival under stress conditions, these findings suggest that impairment of stress-response mechanisms may further contribute to increased tumour cell vulnerability, indicating that HSPs expression could represent a potential tumour-intrinsic early biomarker of treatment response, although clinical validation is still lacking [78]. However, the potential clinical utility of these data will require prospective validation in adequately powered, independent, and preferably multi-institutional patient cohorts undergoing standardized ECT protocols.

### 3.4. Side Effects, Tolerance, and Patient Selection

An important benefit of ECT is its low incidence of treatment-related adverse events [79,80,81]. The most frequently reported side effects are localized, including transient erythema, edema, superficial ulceration, and mild-to-moderate pain at the site of pulse delivery depending on pulse parameters [1,82]. Possible occurrence of localized pain is typically managed effectively with short-term, non-opioid analgesics.Long-term effects are related to excessive electrical or thermal exposure and may include irreversible cell damage and structural tissue injury. High pulse repetition rate protocols have been proposed to reduce neuromuscular stimulation and limit thermal damage, improving the safety profile of the procedure [82]. In a study conducted within the ESOPE framework, the safety and tolerability of ECT were evaluated in patients with cutaneous and subcutaneous tumours treated with BLM or CDDP administered either intravenously or intratumorally, followed by electric pulse delivery. Owing to the low doses of chemotherapeutic agents employed, treatment-related systemic toxicities were minimal, while pain was generally mild and localized to the treated area. However, patients treated under general anaesthesia reported lower pain scores than those receiving local anaesthesia [83]. Similarly, data from a multicentre retrospective study involving patients with advanced skin tumours and cutaneous metastases showed that adverse events associated with ECT were generally manageable and could be classified as systemic or cutaneous [84]. Systemic toxicities, mainly related to anaesthesia or cytostatic drug administration, consisted predominantly of post-procedural nausea and dizziness. Cutaneous adverse events were localized to the treatment area and included superficial and deep necrosis, hyperpigmentation, localized bleeding, and occasional local infections [84]. Furthermore, rare but severe neurological adverse events have been reported. In a case report describing two patients treated with ECT for NMSCs of the head and neck region, one patient with BCC developed post-treatment seizures, whereas another patient with SCC experienced a fatal ischemic stroke [85]. Given the temporal association between treatment and symptom onset, the authors suggested a possible relationship between ECT and the observed neurological complications [85]. Although a direct causal link could not be definitively established, the report highlights the need for careful patient selection and further investigation of potential neurovascular risks, particularly when treating lesions located in anatomically complex and highly vascularized regions [85].

## 4. Electrochemotherapy in Cutaneous Melanoma: Preclinical and Clinical Evidence

Cutaneous melanoma is a highly aggressive malignancy that develops from melanocytes, the melanin-producing cells of the skin [86,87]. Although less common than other skin cancers, it has a high potential to metastatic spread if not diagnosed early [87]. Treatment varies according to stage [88]. Early-stage melanomas are primarily managed with surgical resection, whereas advanced or metastatic disease requires systemic therapies, such as immune checkpoint inhibitors and targeted therapies for BRAF mutations, or, in selected cases, radiotherapy [89,90]. Among locoregional treatments, ECT has emerged as a safe and effective option, providing high local response rates while maintaining minimal systemic toxicity. ECT additionally represents a valuable palliative approach, for patients who are not candidates for surgery or systemic medical therapies [79,91].

Since the early development of electroporation-based therapies, melanoma has been extensively used in in vitro, in vivo animal models, and clinical studies to evaluate the efficacy and safety of ECT.

### 4.1. In Vitro Evidence

In contrast to the extensive clinical evidence supporting ECT in melanoma, relatively few in vitro studies have been conducted using melanoma cell lines. In a study conducted on murine cutaneous melanoma B16-F1 cells it has been demonstrated that the application of high-frequency bipolar pulses in combination with CDDP resulted in greater cytotoxic efficacy compared to either CDDP or electric pulses alone, while potentially reducing muscle contractions and associated pain [92]. Optimization of pulsed electric field parameters, such as pulse width and intensity, is critical for maximizing selective cytotoxicity against tumour cells while preserving normal skin cells. Comparative studies on melanoma and normal skin cell lines have demonstrated that tumour cells are generally more sensitive to electric fields, and that careful tuning of pulse characteristics can enhance antitumor efficacy and improve the safety profile of ECT [93]. The study by Kesar and collaborators observed that ECT triggers a significant time-dependent release of immunologically crucial factors such as ATP, which varies according to drug type (BLM, CDDP and oxaliplatin, OXA) and concentration. The immunogenic response, including alterations in MHC-I and PD-L1 expression, is heavily dependent on the specific chemotherapeutic agent and dosage used. Notably, the OXA-based ECT elicited the most robust immunostimulatory modifications across the tested murine cell lines [53]. Interestingly, while ECT electrical setting per se exerted similar cytoxicity across all cell lines, B16-F10 metastatic melanoma cells appeared slightly more resistant to OXA as compared to other tumour type as 4T1 (breast) and CT26 (colon) cancer cells [53]. Detailed kinetic analysis revealed that a substantial percentage of cells die immediately after treatment, whereas the surviving population remains in cell cycle arrest for at least 24 h. This indicates the existence of two distinct peaks of cell death in vitro: the first occurs immediately post-ECT and is likely driven by membrane perturbations; the second one manifests between 24 and 48 h, driven by intracellular cytotoxic action of massive, delivered drug [53]. Ultimately, the modified expression of key surface markers, including MHC I, MHC II, PD-L1 and CD40, confirmed that ECT acts as a critical enabler in modulating the tumour microenvironment to enhance immune recognition [53]. Another study comparing CDDP and OXA in B16-F10 cells confirmed comparable results with both drugs in terms of direct cytotoxicity and immune response following ECT [94].

Subsequent investigations explored the interaction between ECT and targeted therapies. In human melanoma cells, Dolinšek et al. demonstrated that BLM-based ECT remains effective in BRAF-mutated cells and may exert additive or synergistic effects when combined with BRAF inhibitors, suggesting the potential integration of ECT with targeted therapeutic approaches [95]. To better reproduce the structural complexity of melanoma lesions, more advanced in vitro systems, including three-dimensional cultures and tumour spheroids, were later introduced. These models confirmed that ECT significantly suppresses melanoma cell proliferation and induces extensive cell death in both two- and three-dimensional conditions, indicating that the increased drug uptake induced by electroporation translates into enhanced antitumor activity in more physiologically relevant settings [96,97,98,99,100]. Further in vitro studies evaluated alternative chemotherapeutic agents [101,102], such as dacarbazine (DTIC) [103], demonstrating that electroporation can potentiate the cytotoxic effect of different drugs in melanoma cells, thereby supporting the concept that ECT acts as a versatile drug-delivery platform rather than a drug-specific treatment modality.

To assess the potential of DTIC as an electrochemotherapeutic agent, the authors investigated DTIC-based ECT in an in vitro model of human melanoma cells and dermal fibroblasts. After optimizing electroporation parameters, an electric field strength of 1500 V/cm was identified as the best compromise between membrane permeabilization and cell viability. DTIC alone exhibited greater cytotoxicity toward melanoma cells than fibroblasts at lower concentrations, whereas ECT significantly enhanced drug efficacy, reducing the IC50 values by 2.2-fold in melanoma cells and 2.7-fold in dermal fibroblasts compared with DTIC alone [103]. Similarly, betulinic acid (BTA) has been explored as an alternative agent for ECT. Using metastatic melanoma (Me45) and adenocarcinoma (SW626) cell lines, the authors investigated the effects of ECT on cell viability and HSPs expression (HSP27 and HSP70). Electroporation significantly enhanced the cytotoxic activity of BTA in melanoma cells, whereas a less pronounced effect was observed in adenocarcinoma cells, highlighting the tumour type-dependent efficacy of electroporation-based treatments [78].

### 4.2. Evidence from In Vivo Models

Experimental works using murine melanoma models validated that the combination of electric pulses with BLM significantly enhances the local antitumor effect compared with chemotherapy alone, leading to marked tumour regression and prolonged survival, while also allowing the definition of optimal drug doses and electric field parameters required for effective treatment [21,104,105]. Subsequent investigations explored the possibility of combining ECT with gene electro-transfer in mouse melanoma, showing that the association of ECT with cytokine gene delivery could not only induce local tumour regression but also stimulate systemic antitumor immunity and resistance to tumour rechallenge, supporting the immunomodulatory role of electroporation-based therapies [106,107,108]. In one of the most recent studies, CaEP demonstrated dose-dependent antitumor activity both in vitro, using murine melanoma B16-F10 and murine mammary carcinoma 4T1 cell lines, and in vivo. At higher calcium concentrations, CaEP achieved antitumor efficacy comparable to that of BLM-based ECT in the 4T1 tumour model. Notably, treatment responses were tumour type-dependent; in the B16-F10 melanoma model, the addition of peritumoral *IL-12* gene electrotransfer significantly prolonged survival and promoted tumour microenvironment remodelling, characterized by increased CD8^+^ T-cell infiltration and vascular alterations, thereby highlighting the contribution of immune-mediated mechanisms to overall therapeutic efficacy [109]. Subsequent studies focused on optimizing drug delivery and elucidating the mechanisms governing in vivo treatment efficacy. Moreover, the feasibility of a non-invasive, contactless electroporation approach was demonstrated in a murine B16 melanoma model, where pulsed electromagnetic field (PEMF)-mediated delivery of CDDP enhanced drug accumulation within the tumour and produced a significant tumour growth delay comparable to that achieved with conventional ECT, supporting its potential as a painless alternative to standard ECT [110].

### 4.3. Clinical Findings

The efficacy of ECT in melanoma has been confirmed in numerous clinical studies (Table 1) involving patients with cutaneous and subcutaneous metastases dissemination.

Early clinical investigations evaluated electroporation-mediated delivery of cytotoxic agents in patients with melanoma metastases, having as primary objective feasibility, treatment tolerability, and local antitumor activity [111,112,113,114,115]. Electric pulse application to melanoma nodules enhances the antitumor efficacy of CDDP in patients receiving intravenous CDDP and immunotherapy [116]. This strategy serves as an effective adjunct for local tumour control, extending the median time to progression from 4 to 21 weeks, particularly in patients with unresectable cutaneous and lymph node metastases [116]. Other studies focused on technical refinement of the procedure by investigating the impact of drug administration routes, electrical pulse parameters, and lesion characteristics on treatment outcomes [117,118,119]. In a phase II clinical study, intratumoural CDDP administration followed by electric pulse delivery was evaluated in patients with malignant melanoma presenting with cutaneous nodules who had either failed previous therapies or declined conventional treatment options, demonstrating the antitumour potential of this approach [117]. Subsequently, the influence of pulse parameters was further explored by testing different electric field strengths compared with the pioneering phase I-II study by Mir and colleagues in SCC [30]. Specifically, the electric field intensity was reduced from 1300 V/cm to 1000 V/cm to achieve effective electroporation across tumours of varying sizes while improving safety and minimizing adverse effects [118]. With its broader clinical adoption, subsequent prospective and observational real-world studies further evaluated ECT in patients with in-transit or recurrent cutaneous melanoma metastases. Studies investigating patients with advanced cutaneous metastases, including lesions refractory to previous treatments, demonstrated high local response rates with BLM-based ECT, together with a favourable safety profile even in elderly patients. Response rates appeared to be influenced by lesion size, with smaller metastases showing higher complete response (CR) rates, supporting the importance of appropriate patient and lesion selection [120,121]. Furthermore, ECT was also shown to be effective and well tolerated in the management of challenging bleeding skin metastases, where meaningful clinical benefit could be achieved even after a single treatment session [119].

Later clinical series evaluated the largest number of cutaneous melanoma metastases treated with ECT reported at that time. Notably, the study by Quaglino et al., highlighted the feasibility and clinical benefit of repeated ECT sessions, showing that re-treatment could further improve tumour responses and potentially overcome the lower efficacy generally observed in larger metastases (>1 cm^2^) [122]. Consistent with these findings, a single-institution study including 608 superficial metastases from various tumour types, included cutaneous melanoma, unsuitable for conventional therapies assessed the safety and impact on quality of life of BLM-based ECT performed according to the ESOPE guidelines. The authors reported substantial local response rates after the first treatment, with additional clinical improvement following a second ECT session, supporting the role of repeated applications in optimizing therapeutic outcomes while maintaining a favourable tolerability profile [123].

In palliative settings, multicentre experiences including patients with melanoma and other tumour types treated with intravenous BLM highlighted that, the best outcomes were achieved in patients with a limited number of lesions (<10) and smaller nodules (<2 cm), identifying clinical factors associated with improved treatment efficacy [124]. Further single-institution analyses expanded the evidence base, reporting the outcomes of ECT in a large cohort of melanoma patients, including 654 treated lesions. These studies confirmed the effectiveness of ECT, demonstrating durable local tumour control, reduction of symptoms such as pain and spontaneous bleeding, and improvement in patients’ quality of life [125]. Collectively, these studies contributed to the validation, refinement, and broader implementation of standardized ECT procedures originally established by the ESOPE guidelines. Larger prospective cohort analyses further strengthened the evidence supporting ECT in metastatic melanoma. The multicentre InspECT study evaluated the clinical benefit of ECT using either intravenous or intratumoural BLM, with the possibility of repeated treatment sessions when required. The study confirmed that ECT provides effective locoregional tumour control with a favourable safety profile, as no serious treatment-related adverse events were reported. Observed toxicities were generally mild and included local skin reactions, nausea, flu-like symptoms, and lymphoedema. Importantly, tumour size emerged as a significant predictor of treatment response, with smaller lesions (<3 cm) showing higher CR rates, further supporting the role of careful lesion selection in optimizing clinical outcomes [81]. Taken together, these clinical studies document the progressive transition of ECT from an experimental electroporation-based drug-delivery approach to a standardized and widely accepted therapeutic option for patients with cutaneous metastatic melanoma, particularly in the palliative or locoregional setting and in cases where surgery or systemic treatments are not feasible.

### 4.4. Ongoing Clinical Trial

Several ongoing clinical trials (ClinicalTrials.gov) are currently investigating the role of ECT in melanoma and other solid tumours, with particular attention to biological effects, treatment optimization, and extension of the technique to new clinical settings. A prospective interventional study (NCT06388252) is designed to evaluate the effects of ECT on the tumour immune microenvironment in patients with cutaneous melanoma metastases. In this study, patients with multiple cutaneous or subcutaneous lesions undergo ECT with either intravenous BLM or intratumorally CDDP, and tumour samples collected before and after treatment are analysed histologically and immunohistochemically to assess immune infiltrates, vascular changes, and stromal modifications. The primary objective is to determine whether ECT can convert immunologically “cold” metastases into “hot” lesions, potentially enhancing the efficacy of systemic immunotherapy.

A similar prospective interventional study (NCT07404605) focuses on the biological modifications induced by ECT in cutaneous melanoma metastases, with particular emphasis on microenvironmental changes after treatment. The trial includes patients eligible for standard ECT and compares tumour tissue obtained from treated and untreated lesions to characterize immune, cellular, and stromal responses associated with tumour regression. The aim is to better understand the mechanisms underlying treatment response and to identify biomarkers that may predict clinical benefit. Another ongoing investigation (NCT06753136) explores the use of ECT in a broader oncologic setting, including visceral tumours and metastases of different histological types. This monocentric single-arm interventional study evaluates the safety and objective response rate (ORR) of ECT delivered percutaneously or intraoperatively using a medical device–based electroporation system combined with BLM. Although not limited to melanoma, the study aims to expand the indications of ECT beyond cutaneous lesions and to assess its feasibility for deep or unresectable tumour localizations. In addition, recent clinical protocols such as NCT07092410 are investigating ECT within multimodal treatment strategies, including its integration with systemic therapies and its potential immunomodulatory effects. These studies generally adopt prospective, open-label interventional designs and evaluate endpoints such as local response rate, safety, progression-free survival, and changes in immune-related biomarkers, reflecting the current trend toward combining locoregional tumour ablation with systemic immunotherapy in advanced melanoma. Collectively, current clinical trials indicate that research on ECT is moving beyond simple evaluation of local tumour control toward a more translational approach, aimed at understanding the biological mechanisms of response, optimizing treatment parameters, and integrating ECT into modern multidisciplinary management of metastatic melanoma.

## 5. ECT in Non-Melanoma Skin Cancers

Non-melanoma skin cancers, primarily comprising SCC and BCC, represent the most prevalent malignancies worldwide, accounting for approximately one-third of all newly diagnosed cancers annually [150]. Their incidence has been steadily increasing in recent years, largely driven by prolonged ultraviolet (UV) radiation exposure, global warming, and an aging population [151]. The vast majority of SCC and BCC cases present as low-risk tumours that are successfully managed with surgical excision alone [152]. However, in patients with significant comorbidities, advanced age, or lesions located in cosmetically or functionally sensitive anatomical regions (e.g., the midface, periocular, or perioral areas), extensive surgical resection can result in severe disfigurement and functional impairment [143].

Moreover, while standard treatment modalities, such as surgical excision, Mohs micrographic surgery, and radiation therapy, achieve high cure rates in localized, early-stage disease, the management of locally advanced, recurrent, or multiple synchronous lesions presents substantial therapeutic challenges [153]. In such complex clinical scenarios, ECT represents a valuable locoregional treatment option [139].

From a historical perspective, the clinical application of ECT specifically for NMSC patients was pioneered in 1996 by Heller et al., who evaluated its efficacy on primary and metastatic cutaneous malignancies, including BCC. The study demonstrated highly encouraging outcomes, with most patients achieving a complete response and the remainder showing significant partial regression [112]. This foundational work was subsequently advanced in 1998 by further investigations that validated the clinical utility of combining ECT with intratumorally administered BLM, confirming its effectiveness in successfully eradicating cutaneous and subcutaneous lesions of melanoma, BCC and SCC [114].

To date, the literature regarding ECT for SCC and BCC remains largely dominated by clinical studies (Table 1), whereas in vitro investigations utilizing skin cancer-specific cell lines remain relatively scarce.

### 5.1. In Vitro and Vivo Evidence

-**Basal Cell Carcinoma**: Recent in vitro studies have provided crucial insights into the cellular mechanisms driving ECT efficacy in NMSCs [46,154]. Although establishing stable in vitro BCC primary cultures remains challenging due to their strict reliance on stromal support, targeted cellular analyses have highlighted unique physiological vulnerabilities [155]. Notably, BCC cells exhibit distinct membrane Ca^2+^-ATPase expression profiles compared to normal keratinocytes, making them exceptionally susceptible to CaEP [156]. This targetable vulnerability allows CaEP to induce a massive, uncompensated intracellular calcium overload, triggering acute ATP depletion and rapid, tumour-selective necrosis [156,157,158].

To overcome 2D culture limitations, advanced 3D organotypic co-cultures featuring intact extracellular matrix components and in vivo mouse models (such as *Ptch1*-mutated transgenic mice) have been successfully implemented to recapitulate the complex BCC microenvironment [155]. In these translational and animal systems, CaEP not only drives direct intracellular toxicity but also exploits tissue-specific vascular dynamics, inducing acute local ATP depletion and tumour vascular disruption without damaging adjacent healthy skin [158,159]. Collectively, these preclinical findings substantiate the dual cytotoxic and vascular-disrupting mechanisms that underpin ECT efficacy.

-**Squamous Cell Carcinoma**: In SCC models, electroporation exponentially increases the intracellular uptake of conventional BLM [46] and sensitizes cells to novel agents like mitomycin C by inhibiting protective autophagy and promoting apoptosis [160]. One of the few preclinical studies investigating electric pulse-based therapies in SCC employed both SCC-derived cell lines and in vivo models: cutaneous SCC cell lines were used alongside additional cancer models to evaluate the effects of nanosecond pulsed electric fields (nsPEFs) in vitro, demonstrating that nsPEFs exposure effectively induces tumour cell death and promotes tumour regression in murine models [161].

Furthermore, recent preclinical studies utilizing advanced 3D models and in vivo animal systems have provided pivotal insights into the physiological mechanisms underlying ECT efficacy in NMSCs [162,163,164]. In SCC 3D tumour spheroids and xenograft murine models, electroporation markedly enhances the intratumoural accumulation of BLM and CDDP, effectively disrupting 3D cellular architecture, suppressing tumour angiogenesis, and triggering extensive apoptotic cell death [164].

### 5.2. Clinical Findings

-**Basal Cell Carcinoma**: As anticipated before, in 1996 Heller and co-workers performed a phase I/II trial including BCC, metastatic melanoma and breast cancer patients produced encouraging preliminary results concerning clinical response and tolerability [112]. One year later, another study focused on 20 BCC patients carrying multiple lesions and demonstrated complete response in 53 (98%), and in the majority of these (94%) after a single treatment as well as no recurrence in an 18-month period [126].

Another group enrolled recurrent eyelid-periocular BCC for ECT using intratumoural BLM or intravenous administration for patients with multiple tumours, including those with extra-periocular skin localization [127]. This study confirmed high sensitivity of BCC to ECT also in Gorlin-Goltz syndromic patients and xeroderma pigmentosum genetically predisposed subjects. Subsequently, two independent research groups reported positive outcomes in managing case series of Gorlin-Goltz syndrome using BLM-based ECT, with the CR rate reaching 87% of the treated lesions [128,129]. Recent prospective cohort studies indicate that curative ECT for primary, treatment-naive BCC is superior to its application in recurrent, heavily pre-treated lesions, often yielding long-term local control rates comparable to conventional surgery [130].

A comprehensive 10-year experience published by Campana et al. confirmed the high efficacy and safety profile of ECT in patients presenting with local or locally advanced BCC [131]. The findings underscore that ECT provides durable local tumour regression and can be safely repeated, establishing it as a crucial weapon in the therapeutic armamentarium for non-resectable skin cancers [131]. Then, a recent prospective randomized non-inferiority trial evaluating primary BCC treatments, reported comparable outcomes between ECT and the surgical gold standard [132]. Crucially, over a long-term 5-year follow-up period, the non-recurrence rate was sustained at 87.5% in the ECT cohort compared to 97.5% in the surgical arm, establishing ECT as a highly durable, tissue-sparing alternative [132].

In BCC management, ECT represents a highly effective modality for achieving durable disease control and complete tumour clearance, particularly in complex anatomical areas. Inoperable periocular and infraorbital BCC can achieve complete disease resolution through a combination of intratumoral BLM injection and ECT. In the systematic review by Tan and Oh evaluating ECT in keratinocyte carcinomas using RECIST criteria (CR, PR, SD, PD), patients with BCC demonstrated CR rates ranging from 50% to 100%, with PD generally reported as 0% [165]. Tumour dimension remains a key determinant of treatment efficacy: the ORR reached 93.5% in patients with smaller BCC lesions (≤3 cm) compared to 66.7% in those with larger tumours (>3 cm) [165]. Despite size-dependent variability, long-term survival outcomes in BCC remain exceptionally high, with reported OS ranging from 95% at 14 months to 100% at 1 year [165]. Evidence regarding the integration of ECT with targeted therapies is also emerging in advanced BCC, where ECT has been investigated as a complementary or sequential strategy alongside Hedgehog pathway inhibitors (such as vismodegib) to consolidate local responses and optimize disease control [131]. Overall, international multicentre registry data from the Inspira and EURECA projects confirm that ECT yields exceptional local control in NMSCs (including BCC and SCC), with aggregate ORR ranging between 80% and 100% and CR consistently exceeding 70% to 85% following a single application [133].

Within the context of BCC management, patients with Gorlin-Goltz syndrome (Nevoid Basal Cell Carcinoma Syndrome) represent a highly distinct and complex clinical subpopulation. In these genetically predisposed individuals, managing multiple, recurrent BCCs presents an ongoing challenge, particularly because ionizing radiation is strictly contraindicated due to the high risk of inducing secondary malignancies. In this specific scenario, ECT with BLM has emerged as a valuable, tissue-sparing therapeutic strategy. Clinical evidence demonstrates that ECT achieves high local response rates and durable tumour control across multiple synchronous or metachronous BCC lesions. Because ECT relies on a non-ionizing physical mechanism and leaves minimal scarring, it can be safely repeated over time, offering effective local disease control while preserving anatomical structure and mitigating surgical fatigue in this unique patient subset [128,134].

-**Squamous Cell Carcinoma**: In SCC, ECT has proven highly effective in treating both localized primary lesions and painful, bleeding cutaneous metastases secondary to advanced disease progression [135]. A 2024 meta-analysis including 162 patients demonstrated the superiority of ECT in treating primary tumours and small-to-medium skin lesions (≤3 cm) compared to recurrent tumours and lesions larger than 3 cm [166]. Inoperable periocular and infraorbital SCC can achieve complete disease resolution through a combination of intratumorally BLM injection and ECT. Furthermore, for advanced, in-transit metastases or locally destructive tumours, ECT is increasingly utilized as a neoadjuvant, down-sizing tool to reduce tumour volume prior to planned surgical resection, thereby minimizing required surgical margins. Bartolo and collaborators also reported the successful treatment of a case of Epidermolysis Bullosa, a rare genetic skin disease predisposed to SCC onset [136]. Early clinical studies on ECT in NMSCs primarily evaluated systemic BLM [113,137], while subsequent investigations validated intratumorally BLM, achieving high local response rates and eradicating small/medium lesions with minimal toxicity [115,138]. Intralesional CDDP has also demonstrated favourable efficacy and safety, expanding option tools for patients unsuitable for conventional treatments [114,139]. As clinical confidence grew, ECT applications expanded to advanced disease settings [113,114,138,167], showing high rates of local disease control and symptom relief in cutaneous/subcutaneous metastases [115,123,124,140,141]. In recurrent NMSCs, particularly in anatomically challenging locations or heavily pretreated fields (surgery/radiotherapy), ECT provides durable local control with acceptable cosmetic outcomes [142,143]. Long-term clinical data indicate that dose-de-escalated intravenous BLM preserves sustained local control over a 28-month follow-up [144,145]. Optimizing patient quality-of-life led to the InspECT database, which identified factors linked to treatment-related pain [122] and established reduced-dose protocols suitable for frail patients, elderly populations, or those with impaired renal function requiring repeated sessions [145,146,147]. Major validation came from the multicentre EURECA project in head and neck cancer, reporting 1-year OS and disease-free survival rates of 76% and 89%, respectively [133]. Further InspECT analyses confirmed robust cross-histology safety and efficacy [148]. In a systematic review by Tan and Oh evaluating RECIST outcomes, SCC achieved CR rates ranging from 10% to 100%, with PD up to 9.5% in lesions >3 cm and follow-up OS ranging from 64% at 1 year to 85.1% at 8.6 months; ORR was heavily size-dependent (28.6% for >3 cm vs. 76.9% for ≤3 cm) [165]. Finally, combining ECT with immune checkpoint inhibitors (e.g., anti-PD-1 therapy) shows promising synergistic potential by enhancing antitumor immune responses [149].

## 6. Future Prospectives

Future developments in electric field-based oncological therapies focus on two main frontiers: optimizing drug delivery methodologies while mitigating adverse side effects and expanding the repertoire of transmissible therapeutic agents. Regarding the latter, intratumoural gene transfer and the utilization of tumour-specific antigens for cancer vaccines represent areas of paramount interest.

Three trials evaluated electroporated IL-12 plasmids in melanoma to induce a localized pro-inflammatory response and activate systemic adaptive immunity. However, IL-12 gene transfer demonstrated inferior efficacy compared to traditional ECT. Specifically, Daud et al. reported a CR rate of only 9% for injected lesions [168]. In Greaney et al., a paradoxical immune response emerged: while IL-12 successfully boosted local tumour-infiltrating lymphocytes (CD3^+^ cells), it simultaneously triggered a systemic decrease in tumour-reactive PD-1^+^ cells, indicating a failure to propagate a coordinated systemic response [169]. Consequently, when combined with immunotherapy, IL-12 electroporation achieved an ORR of only 48%, significantly underperforming compared to the 77.8% ORR seen in ECT + pembrolizumab trials [170]. Although limited by sample size, these data suggest that IL-12 alone lacks the robust ICD dynamic of ECT.

In a Phase I clinical trial, Yuan et al. [171] investigated the safety and immunogenicity of electroporated DNA vaccination targeting the melanoma-associated antigen tyrosinase via the pING-muTyr plasmid. The study demonstrated a clear dose-dependent immunomodulatory effect; specifically, patients receiving the 1.5 mg dosage exhibited the most significant clinical improvement in the infiltration of CD8^+^ cytotoxic T lymphocytes into the tumour microenvironment. This robust recruitment of effector CD8^+^ T cells highlight the potential of electroporation to overcome local immune tolerance, although its long-term impact on patient survival remains to be validated.

The future paradigm of ECT is rapidly evolving beyond conventional drug encapsulation toward the integration of smart, multi-functional nanotechnologies [172,173,174]. In this context, innovative nanoparticle-based drug delivery systems have been developed, including liposomes, polymeric, dendrimer, micellar, inorganic, carbon-based, lipid, and viral nanocarriers, which can be functionalized with small molecules, peptides, fluorophores, or antibodies for selective tumour targeting [173,174].

Next-generation strategies prioritize the deployment of dual-triggered, stimuli-responsive nanocarriers designed to undergo structural destabilization and payload release exclusively upon exposure to intrinsic or extrinsic cues, such as the electric pulses of ECT [172,175,176]. Nanocarriers preferentially accumulate within the tumour microenvironment via passive or active targeting strategies, thereby escalating the local bioavailability of chemotherapeutic agents prior to permeabilization. When the ECT electric pulse hits the cell, it not only opens the membrane pores but also structurally disrupts the nanoparticle, causing an on-demand, ultra-rapid drug release confined exclusively to the electroporated area, facilitating an enhanced intracellular internalization of the encapsulated payload [172,175]. This synergistic integration of nanotechnology and electroporation represents a highly promising paradigm to optimize the selectivity and efficacy of locoregional anticancer interventions, concurrently mitigating systemic toxicities [174].

Among these platforms, liposomes have emerged as the most clinically advanced system due to their excellent biocompatibility, favourable safety profile, and versatility in encapsulating both hydrophilic and hydrophobic compounds. Accordingly, several liposomal formulations are currently under clinical evaluation for the treatment of head and neck cancer [174]. In line with this approach, a recent preclinical study demonstrated that the combination of irreversible electroporation with a liposomal formulation of a hydrophobic PI3K/mTOR inhibitor (BEZ235) achieved superior tumour control and prevented tumour recurrence in a head and neck cancer xenograft model compared with electroporation alone [177].

Another cutting-edge technology merges ECT with magnetic therapy. This strategy utilizes magnetoelectric core–shell nanoparticles (e.g., CoFe_2_O_4_, BaTiO_3_). These particles exhibit a unique property: when subjected to an external magnetic field, they locally generate a minute electric field directly at the cellular membrane level [178,179]. In the future, this approach could enable “remote electroporation” without the need to physically insert electrode needles into deep-seated tumours. This approach has been validated in vitro on SKOV-3, a human ovarian carcinoma cell line [180].

Besides liposomes, gold nanoparticles (AuNPs) have also attracted considerable interest as multifunctional nanomaterials for electroporation-based therapies because of their biocompatibility and ability to locally enhance electric field effects. In a preclinical in vitro study using human laryngeal carcinoma (Hep-2) cells, AuNPs enhanced the pro-apoptotic effects of electroporation by modulating apoptosis-related genes, leading to increased cancer cell death [181]. Similarly, a recent in vitro study demonstrated that AuNPs locally amplify the electric field near the cell membrane, reducing the threshold for permeabilization. This effect significantly enhanced BLM uptake during ECT and increased cytotoxicity while safely maintaining reversible electroporation conditions [182]. These promising in vitro findings were further supported by the first in vivo study evaluating AuNP-assisted ECT, in which the combination of BLM-based ECT and AuNPs delayed tumour regrowth and reduced lung metastasis in a murine 4T1 triple-negative breast cancer model [176]. Notably, nanoparticle size emerged as a critical determinant of treatment efficacy, emphasizing the importance of optimizing the physicochemical properties of AuNPs to further improve nanotechnology-assisted ECT [176].

Although the combination of nanomedicine with ECT is still largely at the preclinical stage, encouraging results have been reported in other experimental models, such as liver, pancreatic, and bone tumours, highlighting its potential as a future direction for optimizing electroporation-based cancer therapies [173,174,183,184]. Based on the promising findings reported in these tumour models, the integration of nanotechnology with ECT can be considered a compelling therapeutic option for the treatment of melanoma and NMSCs. Their superficial location facilitates the local administration of nanocarriers, allowing direct monitoring of the treatment response. As research continues to advance, nanotechnology-assisted ECT may offer new opportunities to further improve the clinical management of skin cancers. Another promising avenue for future development is the combination of ECT with radiotherapy, which shows strong clinical and biological potential. Preclinical data consistently report enhanced tumour control with this combined approach compared with either monotherapy alone, supporting its role as a complementary locoregional strategy. Although a specific radiosensitizing synergy has not yet been conclusively demonstrated, these findings provide a solid rationale for further exploring ECT-radiotherapy combinations in prospective clinical trials, including their novel application in neoadjuvant settings [185].

## 7. Concluding Remarks

ECT is rapidly acquiring a pivotal role in the management of primary skin tumours as well as cutaneous and dermal metastases originating from extracutaneous malignancies. By leveraging universal mechanisms of cytotoxicity, the therapeutic efficacy of ECT functions largely independently of the precise histological subtype of the target lesion. Beyond melanoma and NMSCs, emerging clinical evidence highlights its therapeutic success in rare and complex cutaneous neoplasms, including adnexal tumours such as cylindroma [186], dermatofibrosarcoma protuberans [187], and Kaposi sarcoma [188]. Taken together, these findings reinforce ECT as a versatile, highly effective, and histology-independent modality in modern dermato-oncology.

However, it is essential to acknowledge that current knowledge regarding these mechanisms remains incomplete. Several key issues must therefore become central focal points for future clinical and translational studies, including:

(i) Treatment protocols and dosing: Defining optimal electric pulse parameters and drug dosing strategies to maximize local control while minimizing patient discomfort.

(ii) Biomarkers and patient selection: Identifying reliable molecular or clinical biomarkers to better select patients who will derive the greatest therapeutic benefit from ECT.

(iii) Combination strategies: Unravelling the synergistic potential of combining ECT with novel systemic agents, particularly immune checkpoint inhibitors and targeted therapies.

Ultimately, deciphering why melanoma, a classical paradigm of high immunogenicity, often exhibits inferior clinical responses to ECT compared to NMSCs remains a critical imperative. Despite triggering core ICD hallmarks, ECT’s translational efficacy for melanoma is highly heterogeneous. Unravelling these mechanistic disparities will be essential to bypass intrinsic resistance and fully harness ICD-inducing strategies in skin oncology.

## Figures and Tables

**Figure 1:**
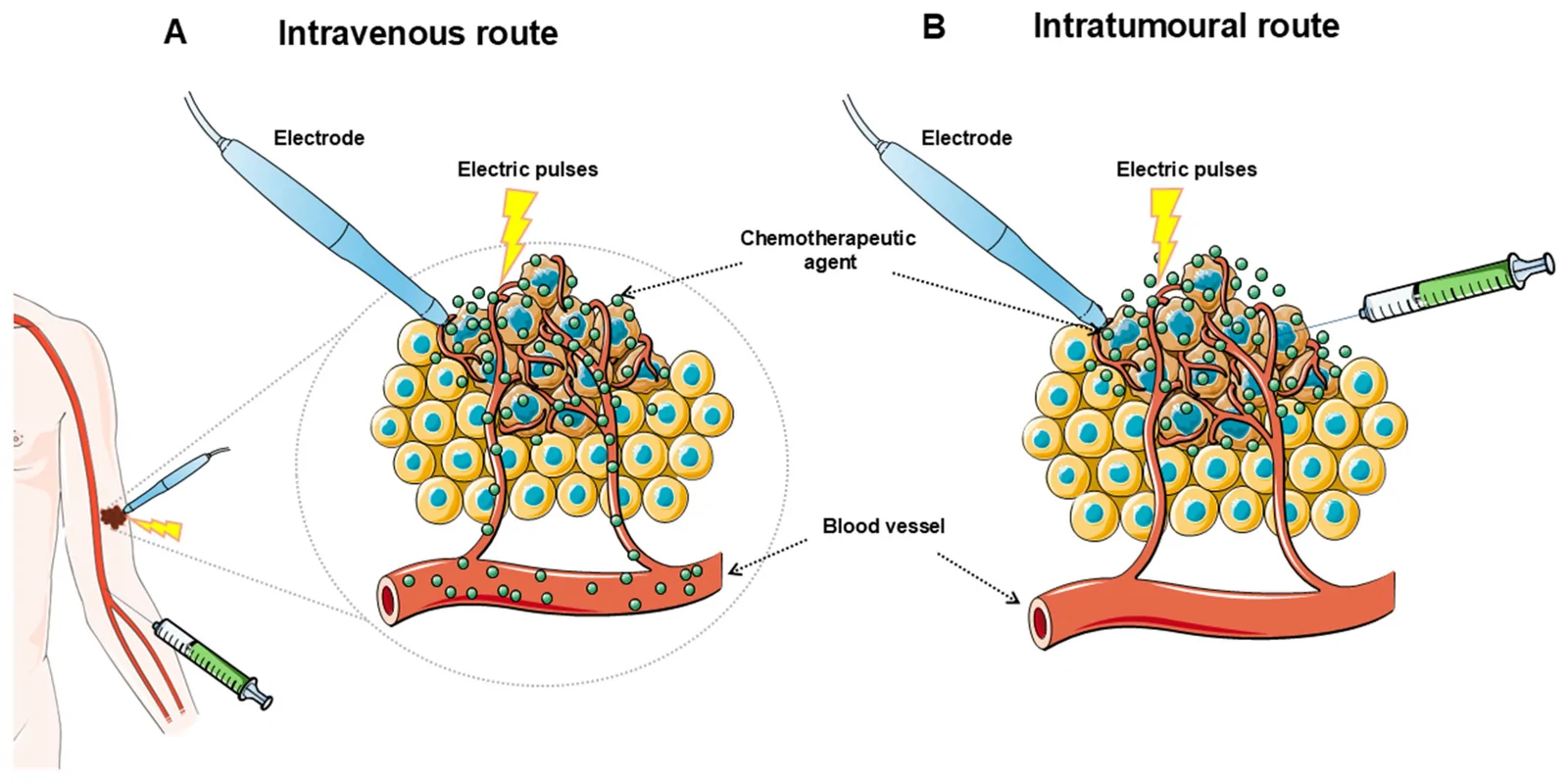
**Routes of chemotherapeutic drug administration in ECT**. Schematic representation of the two administration routes commonly used in ECT. (**A**) Intravenous administration, in which the chemotherapeutic agent is delivered systemically and reaches the tumour through the bloodstream before the application of electric pulses. (**B**) Intratumoural administration, in which the chemotherapeutic agent is injected directly into the tumour lesion, enabling higher intratumoural drug levels with reduced systemic toxicity.

**Figure 2:**
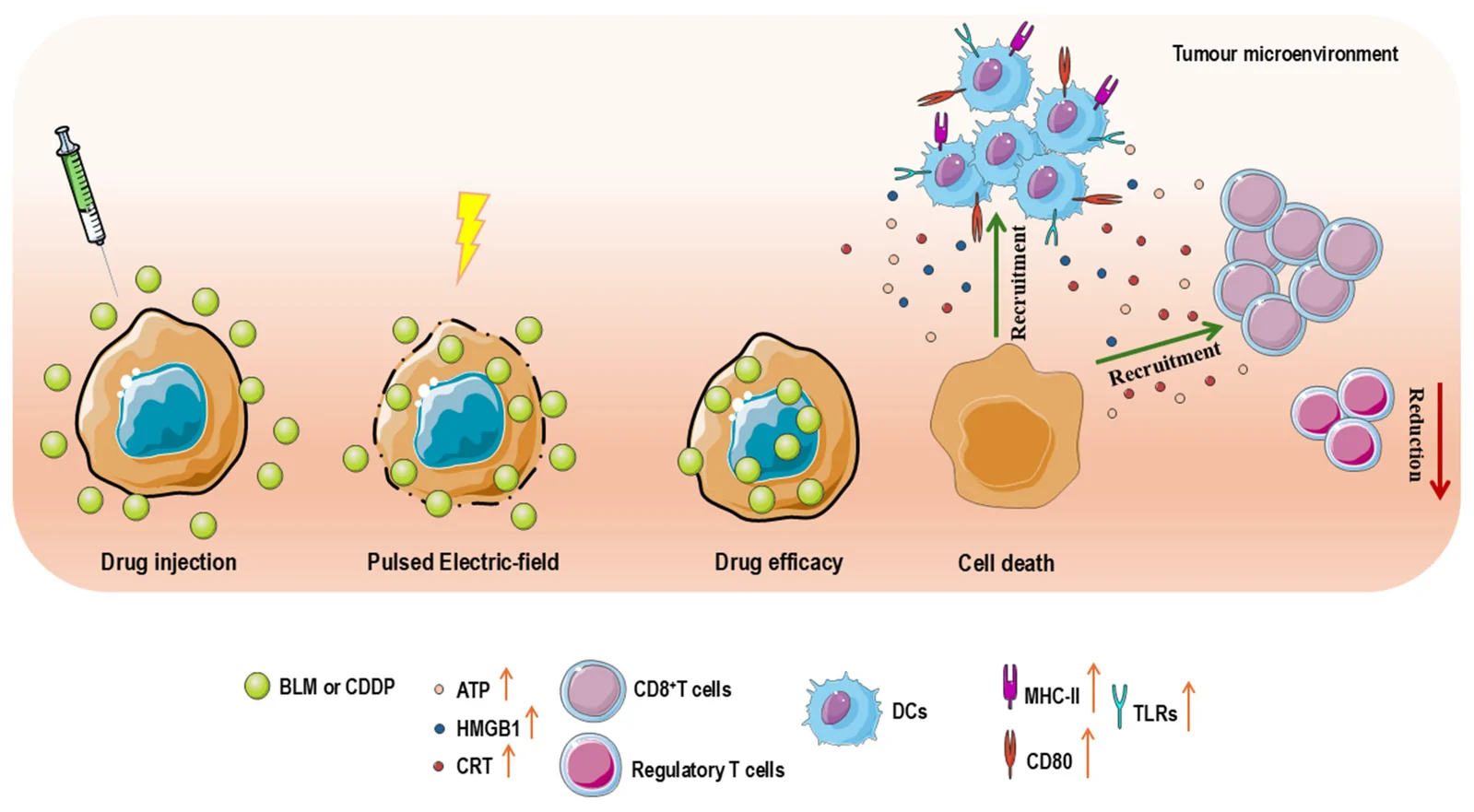
**Immunogenic effects of ECT within the tumour microenvironment.** ECT combines chemotherapeutic drug administration with the delivery of electric pulses, leading to transient membrane permeabilization (electroporation) and enhanced intracellular drug uptake. The increased accumulation of the chemotherapeutic agent induces Immunogenic Cell Death (ICD), resulting in the release and exposure of damage-associated molecular patterns (DAMPs), including adenosine triphosphate (ATP), high-mobility group box 1 (HMGB1) and calreticulin (CRT). These signals promote dendritic cells (DCs) activation and maturation, characterized by increased expression of toll-like receptors (TLRs) and major histocompatibility complex class II (MHC-II), thereby facilitating tumour antigen processing and presentation. Consequently, ECT promotes the recruitment and activation of cytotoxic CD8^+^ T lymphocytes while reducing local immunosuppressive mechanisms, ultimately enhancing antitumour immune responses within the tumour microenvironment.

**Table 1:** Clinical studies of ECT in cutaneous melanoma, SCC and BCC.

Authors (Years)	Study Design	N. of Patients	Tumour Type	Drug	Dose	Admin. Route	Electrode Type
Glass et al. (1996) [111]	Prospective clinical trial	5	Metastatic Melanoma	BLM	0.5–1 U/mm^3^	IT	Plate (caliper) or needle
Heller et al. (1996) [112]	Phase I/II trial	6	Malignant Melanoma/BCC/Metastatic Adenocarcinoma	BLM	10 units/m^2^	IV	Parallel plate
Mir et al. (1998) [113]	Multicentre clinical trial	50	Malignant Melanoma, SCC, BCC, adenocarcinoma	BLM	18–27 units/m^2^ or 0.25–1.0 units/per tumour	IV or IT	Plate (caliper) or needle
Serša et al. (1998) [114]	Prospective clinical trial	4	Metastatic Melanoma, SCC and BCC	CDDP	0.25–2 mg/size tumour	IT	Plate
Rodriguez-Cuevas et al. (2001) [115]	Phase II prospective clinical trial	15	Metastatic Melanoma, SCC and BCC and Breast cancer	BLM	0.5–4 units/m^3^	IT	Needle
Serša et al. (2000) [116]	Phase I-II clinical trial	9	Malignant Melanoma	CDDP + interferon -α2b	20 mg/m^2^ + 3 MIU/m^2^	IV	Parallel
Serša et al. (2000) [117]	Phase II clinical trial	10	Malignant Melanoma	CDDP	0.25–2 mg/size tumour	IT	Parallel
Rols et al. (2000) [118]	Prospective clinical study	4	Malignant Melanoma	BLM	10 mg/m^2^	IV	Parallel
Gehl et al. (2000) [119]	Case report	1	Metastatic Melanoma	BLM	0.15–0.8/mL or 0.08–1.15 U/mm	IV or IT	Needle
Belehradek et al. (1993) [30]	Phase I-II Trial	8	SCC	BLM	10 mg/m^2^	IV	Parallel
Matthiessen et al. (2011) [120]	Non- randomized Phase II multicentre study	52	Malignant Melanoma and Breast cancer	BLM	15,000 IU/m^2^ or IT according to lesion size	IV or IT	Parallel, needle (hexagonal)
Mir-Bonafé et al. (2015) [121]	Retrospective single-center study	31	Metastatic Melanoma	BLM	15,000 IU/m^2^	IV	Hexagonal
Quaglino et al. (2008) [122]	Prospective non- randomized clinical study	14	Metastatic Melanoma	BLM	15 mg/m^2^	IV	Parallel, needle (hexagonal)
Campana et al. (2009) [123]	Prospective non- randomized phase II study	52	Melanoma, Breast cancer, Soft tissue sarcoma, SCC, Head and Neck cancer	BLM	15,000 UI/m^2^ or 1000–250 IU/cm^3^	IV, IT or combined	Parallel and hexagonal
Solari et al. (2014) [124]	Prospective clinical study	39	Melanoma, Breast cancer, Kaposi’s sarcoma, BCC and SCC, Merkel cells carcinoma, Cutaneous Angiosarcoma, and Gastric cell Adenocarcinoma	BLM	15,000 IU/m^2^	IV	Parallel, needle (hexagonal)
Ricotti et al. (2014) [125]	Single-center prospective study	30	Metastatic Melanoma	BLM	15,000 IU/m^2^	IV	NR
Kunte et al. (2017) [81]	Prospective multicentre cohort study (InspECT)	151	Metastatic melanoma	BLM	15,000 IU/m^2^ or 1000 IU/mL	IV or IT	Parallel, needle (hexagonal)
Glass et al. (1997) [126]	Prospective clinical study	20	BCC	BLM	0.5 to 100/mm^3^	IT	Needle
Vass et al. (2024) [127]	Retrospective observational study	19	BCC	BLM	15,000 IU/m^2^ or 250–1000 IU/cm^3^	IV or IT	Needle (linear, hexagonal), plate
Kis et al. (2012) [128]	Case report	3	BCC	BLM	15,000 IU/m^2^	IV	Parallel or needle
Miraglia et al. (2021) [129]	Case report	2	BCC	BLM	15,000 IU/m^2^	IV	Hexagonal needle
Groselj et al. (2025) [130]	Retrospective multicentre cohort study (data from the InspECT registry)	132	BCC	BLM	15,000 IU/m^2^ or max 1000 IU/cm^3^	IV or IT	Needle (linear, hexagonal), plate
Campana et al. (2017) [131]	Retrospective observational study	84	BCC	BLM	15,000 IU/m^2^ or 250–1000 IU/cm^3^	IV or IT	Needle (linear, hexagonal), finger
Clover et al. (2020) [132]	Prospective randomised control trial		BCC	BLM	500 to 5000 IU/cm^3^	IT	Parallel, hexagonal
Bertino et al. (2016) [133]	Multi- institutional prospective study	105	BCC, SCC, Metastatic Melanoma	BLM	15,000 IU/m^2^ or 250–1000 IU/cm^3^	IV or IT	Plate, row-needle (hexagonal) or finger
Rozsa et al. (2025) [134]	Prospective Cohort Analysis	44	BCC, SCC	BLM	15,000 IU/m^2^	IV	Needle (linear, hexagonal)
Caballero-Borrego et al. (2023) [135]	Prospective phase II, observational clinical study	56	SCC, papillary thyroid carcinoma, adenoid cystic carcinoma of parotid gland, Malignant Melanoma	BLM	15–20 mg/m^2^	IV	Needle linear
Bartolo et al. (2020) [136]	Retrospective study	5	SCC	BLM	15,000 IU/m^2^	IV	Needle (hexagonal)
Glass et al. (1996) [137]	Prospective clinical study	2	Nevoid BCC syndrome	BLM	10 U/m^2^	IV	Parallel
Heller et al. (1998) [138]	Clinical study	34	BCC, SCC, Melanoma, Kaposi’s Sarcoma	BLM	0.5 to 4 U/mm^3^	IT	Parallel or needle
De Giorgi et al. (2020) [139]	Single-center prospective study	8	BCC, SCC, Melanoma	CDDP	At least 0.441 mL/ lesion	IT	Linear lamellar, finger or linear
Fantini et al. (2008) [140]	Case report	1	Metastatic BCC with Squamous Differentiation	BLM	250 to 1000 IU/cm^3^	IT	Needle (hexagonal)
Campana et al. (2016) [141]	Prospective, multicentre, single-arm, investigator-led observational cohort study	376	Melanoma, BCC, SCC, Kaposi’s sarcoma, Soft tissue sarcoma, Breast carcinoma	BLM; CDDP	15,000 IU/m^2^ or 250–1000 IU/cm^3^; 0.5–2 mg/cm^3^	IV or IT; IT	Needle (linear, hexagonal), plate
Mevio et al. (2012) [142]	Prospective clinical trial	15	SCC, Basaloid carcinoma and Merkel cell carcinoma	BLM	15,000 IU/m^2^	IV	Plate, hexagonal, or finger
Yazdan Parast et al. (2025) [143]	Single-arm phase 2 clinical trial	26	BCC, SCC, Melanoma	BLM	15,000 IU/m^2^	IV	Parallel, needle (hexagonal)
Kristiansson et al. (2019) [144]	Retrospective observational cohort study	7	SCC, BCC	BLM	1000 IU/cm^3^	IT	Needle hexagonal
Jamsek et al. (2020) [145]	Non- randomized prospective study	28	NMSCs	BLM	10,000 IU/m^2^ vs. 15,000 IU/m^2^	IV	Hexagonal, needle row, or plate
Groselj et al. (2018) [146]	Non- randomized prospective controlled study	28	BCC, SCC	BLM	10,000 IU/m^2^ vs. 15,000 IU/m^2^	IV	Hexagonal, needle row, or plate
Rotunno et al. (2018) [147]	Retrospective analysis of prospectively collected data (InspECT registry)	57	BCC, SCC, Melanoma, Breast cancer, Kaposi’s sarcoma	BLM	7500–10,000–13,500 IU/m^2^	IV	Plate, Needles (linear, hexagonal)
Clover et al. (2020) [148]	Multicentre prospective registry analysis (InspECT database)	987	Metastatic Melanoma, SCC, BCC, Kaposi’s Sarcoma, Breast cancer	BLM	15,000 IU/m^2^ or 1000 IU/cm^3^	IV or IT	Plate, row-needle, hexagonal
Barca et al. (2023) [149]	Case report	1	SCC	BLM + cemi- plimab	NR	IV	Parallel, needle (linear, hexagonal)

BCC = Basal Cell Carcinoma; BLM = Bleomycin; CDDP =Cisplatin; IV = intravenous; IT = intratumoural; N. = Number; NR = Not Reported; SCC = Squamous Cell Carcinoma.

## Data Availability

No new data were created or analyzed in this study. Data sharing is not applicable to this article.
